# Preemptive ECMO Cannulation in a Patient with Severe Left Ventricular Systolic Dysfunction Undergoing Cesarean Delivery

**DOI:** 10.1155/2019/2623608

**Published:** 2019-04-08

**Authors:** Phuong Bui, P. Gabriel Mascaro, Juanita Henao-Mejia, Selina Patel

**Affiliations:** ^1^Brigham and Women's Hospital, Department of Anesthesiology and Perioperative and Pain Medicine, 75 Francis Street, Boston, MA 02115, USA; ^2^University of Miami, Miller School of Medicine, Department of Anesthesiology, Perioperative Medicine and Pain Management, 1611 NW 12th Ave, C-301, Miami, FL 33136, USA; ^3^The University of Oklahoma Health Sciences Center, Department of Anesthesiology, 700 NE 13th Street, Oklahoma City, OK 73104, USA

## Abstract

Obstetric patients with heart failure undergoing cesarean delivery are high risk and the perioperative care of these patients poses significant multidisciplinary challenges. In contrast to the nonobstetric patient population the potential role of mechanical circulatory support in parturients with heart failure is not well established and the use of extracorporeal membrane oxygenation (ECMO) has rarely been reported. We report the case of a super morbidly obese patient with decompensated heart failure, pulmonary hypertension, and superimposed preeclampsia undergoing preemptive ECMO cannulation for urgent cesarean delivery.

## 1. Introduction

Cardiovascular disease is the leading cause of maternal mortality in the United States [1]. Heart failure is responsible for over 9% of in-hospital deaths among pregnancy-related hospitalizations and is associated with numerous adverse outcomes [2].

Patients with heart failure may appear clinically stable, but may rapidly decompensate with the dynamic physiological changes and demands of pregnancy, delivery, and the postpartum period. Providing safe perioperative care for these high-risk parturients undergoing cesarean delivery is a complex task and requires thoughtful planning. In a decompensating patient maximal pharmacological therapy itself may not suffice to maintain hemodynamic stability and the institution of mechanical circulatory support may be life-saving.

In the nonobstetric population extracorporeal membrane oxygenation (ECMO) is an established treatment modality in the setting of refractory cardiogenic shock or respiratory failure or as a rescue strategy after cardiac arrest. It provides temporary circulatory and/or respiratory support until recovery or serves as a bridge to long-term mechanical circulatory support or transplantation [3]. The indications for ECMO and its use in nonobstetric patients are well delineated within existing guidelines [4]. In obstetric patients however, experience with ECMO is much more limited and its use has been described in a number of case reports and case series only.

We describe the use of preemptive ECMO cannulation for urgent cesarean delivery in a super morbidly obese parturient with decompensated heart failure, pulmonary hypertension, and superimposed preeclampsia.

## 2. Case Presentation

A 34-year-old gravida 2 para 1 at 25 weeks gestation was transferred to our institution from an outside hospital. She complained of a two-week history of progressively worsening orthopnea and shortness of breath that left her unable to perform daily activities. An initial transthoracic echocardiogram obtained at the referring hospital had demonstrated a depressed ejection fraction (35%) as well as mild pulmonary hypertension. The patient was transferred to our institution for further evaluation and management.

Her past medical history was significant for chronic hypertension, class F diabetes mellitus, super morbid obesity with a BMI of 53, and chronic kidney disease. Two years before, she underwent an emergent cesarean delivery at 35 weeks gestation due to preeclampsia with severe features and nonreassuring fetal status. The patient had no previous history of congenital, ischemic, or valvular heart disease. She had no family history of heart disease and never had an echocardiogram before. On admission she required 2 L/min of oxygen per nasal cannula to maintain an oxygen saturation of 95%. The remaining vital signs were within normal limits. Notable findings on the physical exam were presence of S3 and S4 heart sounds, positive jugular venous distention, bilateral crackles on auscultation, and 2+ pitting edema of the lower extremities. A baseline electrocardiogram revealed normal sinus rhythm. Chest x-ray demonstrated pulmonary interstitial edema and bilateral pleural effusions. A transthoracic echocardiogram demonstrated a moderately dilated left ventricle, eccentric left ventricular hypertrophy, and a severely reduced left ventricular systolic function with an ejection fraction of 20-25% as well as global left ventricular hypokinesis. Mitral inflow pattern and tissue doppler were indicative of grade 3 diastolic dysfunction. The right ventricular function was mildly to moderately reduced. The right ventricular systolic pressure was elevated at 50-60 mmHg and there was a moderate degree of pulmonary hypertension. Complete metabolic profile was significant for hyponatremia of 132 mmol/L, hyperkalemia of 5.6 mmol/L, elevated blood urea nitrogen of 35 mg/dL, creatinine of 2.0 mg/dl with an eGFR of 29 ml/min/1.73m(2), and proBNP of 13000 pg/ml. Arterial blood gas analysis showed a significant metabolic acidosis (base excess -10 mmol/L) with partial respiratory compensation. The complete blood count, hepatic function, coagulation, and thyroid hormone panels were normal. Toxicology screen and screen for HIV, syphilis, and hepatitis were negative. Fetal ultrasound showed an intrauterine singleton pregnancy with suspected intrauterine growth retardation and a fetal heart rate of 140 beats per minute. Continuous fetal monitoring via cardiotocography was impossible due to maternal body habitus.

The patient's overall presentation was consistent with acutely decompensated biventricular systolic and diastolic heart failure. Additionally, multiple severe range blood pressure readings were recorded after admission and the patient was diagnosed with superimposed preeclampsia with severe features including systolic blood pressures >160 mmHg and doubling of baseline creatinine. She was started on magnesium sulfate for seizure prophylaxis and a nitroglycerine infusion for blood pressure control. A furosemide infusion was commenced to correct the patient's pulmonary edema and volume overloaded state. Serial preeclampsia labs were drawn every 6 hours to monitor disease progression. Antenatal betamethasone was administered for fetal lung maturation. Due to the acuity of the patient's condition and complex clinical presentation an urgent multidisciplinary meeting was arranged to discuss further plans of care. Specialties present included obstetrics, obstetric anesthesiology, cardiology, cardiac surgery, cardiac anesthesiology, obstetric nursing, transfusion medicine, maternal fetal medicine, and neonatal intensive care.

In light of her rapidly declining functional status due to exacerbated heart failure combined with superimposed severe preeclampsia, it was agreed that she was not a candidate for expectant management and cesarean delivery would be pursued as soon as possible. Due to her worsening cardiac status it was decided to transfer the patient to the cardiac surgery intensive care unit (ICU) for continuous, invasive hemodynamic monitoring, medical optimization, and completion of antenatal steroids prior to undergoing cesarean delivery. Since she was high risk for cardiovascular decompensation upon induction of anesthesia the cardiac surgical team would be present in the operating room and preinduction femoral cannulas would be inserted for emergent venoarterial ECMO institution in the event of hemodynamic collapse.

In the ICU, a right radial arterial line and right internal jugular vein 9 French introducer catheter were placed. A pulmonary artery catheter was inserted. The cardiac output, pulmonary artery pressure, and pulmonary capillary wedge pressure (PCWP) were 6 l/min, 45/25 mmHg, and 20 mmHg, respectively. Despite medical management with a furosemide infusion, maternal condition continued to decline with worsening renal function (creatinine rise from 1.95 mg/dl to 2.59 mg/dl), hyperkalemia, and increasing oxygen requirements. A left internal jugular hemodialysis catheter was inserted and the patient was placed on continuous veno-venous hemofiltration. The goal was to achieve and maintain euvolemia with a central venous pressure and PCWP of less than 12 mmHg. Further management included blood pressure control with a nitroglycerin infusion and an insulin infusion for glycemic control. She remained hemodynamically stable not requiring inotropic or mechanical circulatory support. After 24 hours of medical optimization in the ICU the patient's condition had improved significantly with correction of volume status and satisfactory end-organ perfusion. She was brought to the operating room for repeat cesarean delivery and bilateral tubal ligation.

The patient was premedicated with sodium citrate/citric acid 30 mL liquid PO and metoclopramide 10 mg intravenously (IV). Standard ASA monitors were attached. Invasive blood pressure, central venous pressure, and pulmonary artery pressure were continuously monitored throughout the case. The cardiac surgical team inserted 7 French introducer cannulas into the right common femoral artery and vein under local anesthesia in order to facilitate emergency insertion of ECMO cannulas if needed. After preoxygenation a modified rapid sequence induction was performed using IV etomidate 16 mg, lidocaine 100 mg, remifentanil 80 mcg, and succinylcholine 140 mg. Intubation was successful on first attempt via video-laryngoscope and a size 7 endotracheal tube was inserted. General anesthesia was maintained with sevoflurane, nitrous oxide, and oxygen and was titrated to maintain a bispectral index between 40 and 60. Transesophageal echocardiography (TEE) revealed a dilated left ventricle and an ejection fraction of <25% with severe global hypokinesis. The right ventricle was dilated and mildly hypokinetic. There was grade 3 diastolic dysfunction. The patient remained hemodynamically stable after induction until the baby was delivered 2 minutes after uterine incision. It was intubated and transferred to the neonatal intensive care unit (APGAR 1/5/7). Immediately on delivery, an infusion of oxytocin at 20 units/h was commenced. However, the patient's uterus remained atonic requiring 1000 mcg misoprostol sublingually, 250 mcg carboprost intramuscularly, and 250 mcg carboprost intrauteral. Bimanual compression was held and uterine tone improved thereafter with total estimated blood loss of 1500 ml. Perioperatively, the patient's vital signs included a heart rate 90-112 bpm, blood pressure 128-89/79-54 mmHg, oxygen saturation 92-100%, central venous pressure 12-17 mmHg, pulmonary artery pressure 50-33/36-22 mmHg, and mean pulmonary artery pressure 25-38 mmHg with most of the hemodynamic variation seen after delivery of the fetus. We attributed these hemodynamic changes to a combination of maternal autotransfusion, maternal hemorrhage, and vasodilation associated with the oxytocin infusion. Fluid resuscitation involved 250 ml crystalloid and 250 ml albumin without blood transfusion. The patient was started on infusions of epinephrine (up to 7 mcg/min for inotropic support), norepinephrine (up to 2 mcg/min for pressor support), and milrinone (up to 0.3 mcg/kg/min for inotropic support and reduction of pulmonary vascular resistance) which were titrated to maintain hemodynamic stability and guided by pulmonary artery catheter and TEE monitoring. The cardiac surgeons were present throughout the entire case in the event ECMO needed to be initiated.

Postoperatively the patient was transferred to the surgical ICU intubated on dexmedetomidine 0.5 mcg/kg/h for sedation as well as oxytocin 2.5 units/h. Milrinone, epinephrine, and norepinephrine infusions were continued. The femoral introducer cannulas were left in situ. In the ICU she remained hemodynamically stable and vasopressor/inotropic support was gradually weaned off. She was extubated on postoperative day (POD) 1. CVVH was discontinued and the patient was diuresed with furosemide. Her kidney function recovered to baseline function. There was no evidence of end-organ hypoperfusion. Her cardiac output was 5 l/min without inotropic support. She was decannulated on POD 2, transferred to the telemetry floor on POD 4, and discharged home on POD 8 on guideline-directed medical therapy for heart failure.

## 3. Discussion

Maternal heart failure occurs due to functional or structural heart disease or a combination of both [5]. A detailed history and examination along with comprehensive diagnostic studies can often aid in identifying the underlying etiology. The differential diagnoses in our case included peripartum cardiomyopathy, preexisting dilated cardiomyopathy, and hypertensive heart disease.

Based on the patient's history, clinical course, and the results of the echocardiogram our multidisciplinary team of physicians suspected an early onset of peripartum cardiomyopathy, an entity exclusively related to pregnancy with an incidence ranging from 1 in 1000 to 4000 live births in the United States [6]. Even though the disease is still relatively uncommon its incidence is rising and its impact on maternal-fetal morbidity and mortality is significant [7]. Although no universally accepted definition exists the Heart Failure Association of the European Society of Cardiology Working Group on Peripartum Cardiomyopathy defines peripartum cardiomyopathy as an idiopathic form of cardiomyopathy with an ejection fraction of less than 45% presenting towards the end of pregnancy or following delivery. The pathophysiological mechanisms are not fully understood and it is hypothesized that increased oxidative stress, inflammation, viral infection, and autoimmune responses may be involved. Notably it is a diagnosis of exclusion when no other definitive cause of heart failure can be identified [8].

Our patient had reached the end of the second trimester which is an early time for diagnosis. However, the onset of peripartum cardiomyopathy may not be exclusively confined to the months immediately before and after delivery. Elkayam et al. compared 123 women with early and late onset pregnancy-associated cardiomyopathy. In this study the earliest gestational age at time of diagnosis was 17 weeks. Since presentation and outcome were similar between the early and late onset groups the authors suggested that they represent a spectrum of the same disease [9].

Although our patient had never experienced previous symptoms of heart failure preexisting cardiomyopathy was certainly possible as well. The clinical presentations of dilated cardiomyopathy and peripartum cardiomyopathy are similar and they may also share echocardiographic key features of left ventricular dilation and systolic dysfunction [10]. As in this case a clear distinction is often times not possible without reviewing a prior echocardiogram. Since our patient had not undergone any previous cardiac imaging her definitive diagnosis ultimately remained uncertain. Her significant diastolic heart failure was most likely attributable to superimposed hypertensive heart disease. In fact a strong association between preeclampsia and peripartum cardiomyopathy has been described. A meta-analysis has found an estimated prevalence of preeclampsia in patients with peripartum cardiomyopathy of 22% [11].

Heart failure management strategies are similar to those for nonpregnant patients and should follow current guidelines. Therapy is aimed towards symptomatic relief and hemodynamic optimization. While inotropes, diuretics, beta blockers, nitrates, and hydralazine can be used safely in the pregnant patient, angiotensin converting enzyme inhibitors and angiotensin II receptor blockers are contraindicated due to fetal toxicity [12].

Patients with refractory heart failure despite maximal medical therapy may require mechanical circulatory support. There are no evidence-based guidelines for mechanical circulatory assist device utilization in pregnancy and the existing data is derived from case reports.

The use of ECMO in obstetric patients during pregnancy, delivery, and the postpartum period has been described in cases of ARDS during the H1N1 influenza pandemic, amniotic fluid embolism, pulmonary embolism, postpartum hemorrhage, and cardiogenic shock [13].

ECMO has been utilized in the setting of peripartum cardiomyopathy. One case describes a 34-year-old at term gestation who presented with acutely worsening heart failure and cesarean delivery was performed after full flow ECMO support was established. ECMO was maintained for five days after surgery [14]. In another case an 18-year-old woman developed decompensated heart failure immediately after cesarean delivery and was placed on ECMO as a bridge to recovery. She was weaned off after twenty-eight hours [15].

Preemptive ECMO cannulation as a stand-by modality in pregnant patients has been described very rarely. Existing reports include a 40-year-old patient with methamphetamine-associated cardiomyopathy undergoing dilation and evacuation and a 39-year-old woman with noncompaction cardiomyopathy undergoing elective cesarean delivery [16, 17].

Apart from ECMO, the use of other percutaneous mechanical circulatory assist devices such as intra-aortic balloon pump (IABP) and Impella has also been described for the management of cardiogenic shock in obstetric patients [18, 19]. Currently, there is insufficient data comparing these modalities for the management of peripartum cardiomyopathy and clinicians should select an appropriate device taking into account the required cardiovascular support (RV versus LV versus both), the oxygenation of the patient, periprocedural risks, and the local expertise available [20]. An IABP provides less mechanical support compared to other devices, but is relatively simple to place and requires less strict anticoagulation. The Impella device provides a high degree of LV support and is effective in LV unloading, but requires good RV function or an RV assist device to ensure adequate preload. Furthermore, Impella placement requires fluoroscopy or echocardiographic guidance. We decided to use ECMO as a backup mechanical assist modality in this patient based on institutional experience, the presence of biventricular failure in our patient, and ability of ECMO to provide maximal cardiovascular support and improvement in oxygenation.

Besides choosing the appropriate mechanical support device, other clinical decisions that needed to be made in our case were timing of cannulation and choice of anesthetic technique. Insertion of peripheral cannulas to facilitate potential ECMO initiation can occur before or after induction of anesthesia. We decided to place femoral cannulas under local anesthesia prior to induction since our patient had biventricular failure with severely reduced left ventricular function and the risk of hemodynamic instability upon induction of general anesthesia was significant. Additionally, our patient's body habitus (BMI 53) could have made vascular cannulation challenging and time consuming. Obviously such technical challenges are best dealt with in a controlled situation versus an emergency. Inability to rapidly establish vascular access for ECMO after cardiovascular collapse could have been catastrophic.

We chose to administer general anesthesia for multiple reasons. Our patient suffered from pulmonary hypertension with moderate right ventricular dysfunction. Mechanical ventilation allowed us to control oxygenation and ventilation. Hypoxemia and hypercapnia which could have further increased pulmonary vasculature resistance were thus prevented. General anesthesia also allowed us to place a TEE probe immediately after induction and the TEE findings provided real time monitoring of cardiac function and guidance for fluid administration and inotropic and vasopressor therapy. Additionally, our patient suffered from orthopnea and her ability to lie supine while awake for a prolonged period of time was doubtful. Lastly the risk of an epidural hematoma after neuraxial anesthesia would have been increased with the administration of large doses of systemic anticoagulants necessary for ECMO institution. Even though we decided to perform general anesthesia for the aforementioned reasons, regional anesthesia has been used successfully in patients with heart failure undergoing cesarean delivery. Specific advantages of regional anesthesia in the setting of heart failure include a decrease in pre- and afterload through sympathectomy, ability to titrate anesthetic medications through a neuraxial catheter ensuring stable hemodynamics, and avoidance of direct cardio-depressant intravenous anesthetic agents. Regional anesthesia also obviates the need to manipulate the airway in a patient population that is at high risk for aspiration and well known for an increased incidence of difficult intubation. Furthermore, neuraxial anesthesia is associated with less maternal blood loss when compared to general anesthesia [21].

Regardless of the chosen anesthetic technique continuous monitoring, meticulous hemodynamic control, and stringent communication are critical for maternal and fetal outcome.

Postoperatively our patient returned to the intensive care unit for further care. Due to the expected physiological changes of the immediate postpartum period the femoral cannulas were left in situ for 48 hours. A team of obstetricians, cardiologists, and nephrologists continued to follow her for comprehensive postoperative care until discharge.

In summary, obstetric patients with heart failure presenting for cesarean delivery pose substantial challenges to all specialties involved. This case report highlights the utility of preemptive ECMO cannulation as a potentially life-saving measure in the perioperative setting. It also illustrates the extensive resources required and the importance of a multidisciplinary approach when caring for these high-risk parturients. The paucity of published data and the absence of evidence-based protocols indicate a need for further research regarding the role of ECMO in the pregnant population.
